# Postoperative circulating tumor DNA detection and CBLB mutations are prognostic biomarkers for gastric cancer

**DOI:** 10.1007/s13258-023-01412-7

**Published:** 2023-06-12

**Authors:** Hekai Zhou, Houcong Liu, Jun Li, Jidong Wang, Xiaohong Fu, Yingqiang Li, Shaolong Mao, Jihui Du

**Affiliations:** 1 Research Center for Clinical and Translational Medicine, Huazhong University of Science and Technology Union Shenzhen Hospital, and the 6th Affiliated Hospital of Shenzhen University Medical School, Guangdong 518052 Shenzhen, China; 2 HaploX Biotechnology Co., Ltd., Shenzhen, 518057 Guangdong China; 3 Department of Oncology, Huazhong University of Science and Technology Union Shenzhen Hospital, and the 6th Affiliated Hospital of Shenzhen University Medical School, Guangdong 518052 Shenzhen, China

**Keywords:** Gastric cancer, ctDNA, Prognosis, CBLB, Biomarker

## Abstract

**Background:**

Several studies have demonstrated that circulating tumor DNA (ctDNA) can be used to predict the postoperative recurrence of several cancers. However, there are few studies on the use of ctDNA as a prognosis tool for gastric cancer (GC) patients.

**Objective:**

This study aims to determine whether ctDNA could be used as a prognostic biomarker in GC patients through multigene-panel sequencing.

**Methods:**

Using next-generation sequencing (NGS) Multigene Panels, the mutational signatures associated with the prognosis of GC patients were identified. We calculated the survival probability with Kaplan–Meier and used the Log-rank test to compare survival curves between ctDNA-positive and ctDNA-negative groups. Potential application of radiology combined with tumor plasma biomarker analysis of ctDNA in GC patients was carried out.

**Results:**

Disease progression is more likely in ctDNA-positive patients as characterized clinically by a generally higher T stage and a poorer therapeutic response (P < 0.05). ctDNA-positive patients also had worse overall-survival (OS: P = 0.203) and progression-free survival (PFS: P = 0.037). The combined analysis of ctDNA, radiological, and serum biomarkers in four patients indicated that ctDNA monitoring can be a good complement to radiological and plasma tumor markers for GC patients. Kaplan–Meier analysis using a cohort of GC patients in the TCGA database showed that patients with CBLB mutations had shorter OS and PFS than wild-type patients (OS: P = 0.0036; PFS: P = 0.0027).

**Conclusions:**

This study confirmed the utility and feasibility of ctDNA in the prognosis monitoring of gastric cancer.

**Supplementary Information:**

The online version contains supplementary material available at 10.1007/s13258-023-01412-7.

## Introduction

Globally, gastric cancer (GC) is estimated to be the fifth most common malignancy with one million new cases every year (Smyth et al. 2020). Because of its advanced stage at diagnosis, GC is the fourth leading cancer-related cause of death worldwide, with approximately 769,000 deaths in 2020 (Sung et al. 2021). With combination cytotoxic chemotherapy, which is the standard of care for patients with unresectable GC, the median OS is only 9–11 months (Allemani et al. 2015). The lack of effective GC screening methods and reliable prognostic biomarkers could be one of the reasons for the low GC survival rate (Joshi and Badgwell 2021). Therefore, there is an urgent need to develop effective diagnostic and prognostic biomarkers for GC.

Cell-free DNA (cfDNA) is the extracellular DNA in the plasma. Circulating tumor DNA (ctDNA) is the cfDNA that is derived from tumor tissue, which can be used as a biomarker for tumor mutations (Keller et al. 2021). Due to intra-tumor heterogeneity, the detection of gene mutation from ctDNA has clear advantages over a single tumor biopsy sample by covering tumor mutations from different subclones. Kim et al. monitored the ctDNA on cancer-specific rearrangements after gastric cancer surgery by WGS analysis and found personalized cancer-specific rearrangements in 19 of 25 gastric cancers. GC recurrence within one year of surgery is associated with postoperative ctDNA presence (Kim et al. 2019). In a prospective cohort study, ctDNA levels detectable by targeted deep sequencing correlated with postoperative disease-free survival (DFS) and OS in patients with GC, indicating that a ctDNA assay can be used for prognosis in gastric cancer (Yang et al. 2020). Another study of the ctDNA from 40 patients with malignant recurrent high grade serous ovarian cancer showed that the patients with TP53 mutation allele score with more than 60% reduction had longer relapse-free survival after one course of chemotherapy (Parkinson et al. 2016). A recent study demonstrated that longitudinal ctDNA sequencing could be used to identify genes responsible for trastuzumab resistance in metastatic HER2-positive GC (Zhang et al. 2020). However, the molecular mutation characteristics of ctDNA and the clinical utility of ctDNA as a predictor of tumor progression in GC have not been fully explored.

The NGS panel with 680 tumor-associated genes was used in the detection of the driver mutations, gene mutations for targeted therapy, and biomarkers for immunotherapy, as well as tracking dynamic changes of ctDNA during therapy (Chen et al. 2020). By analyzing the ctDNA mutations of our patients and patient data from TCGA, we sought to identify significant associations between ctDNA gene mutations and GC progression and to identify the potential ctDNA prognosis biomarkers.

## Materials and methods

### Patients and samples

Fourteen patients pathologically diagnosed with gastric cancer were recruited to the Oncology Center, from March 2019 to October 2021. Samples of peripheral blood were collected from each patient before adjuvant chemotherapy and 4–6 weeks after surgery for sequencing analysis. During follow-up, blood was drawn on 5 patients at multiple times thereafter. The patients were followed up every 3–6 months postoperatively. Each follow-up assessment included physical examination, blood routine test, serum tumor markers (e.g., CEA, CA125, CA19-9) detection, and abdominal CT scan. A description of the baseline characteristics of these patients can be found in Table 1. The ctDNA analysis was performed on 20 blood samples.

**Table 1:** Clinical characteristics of 14 GC patients detected by ctDNA

Variables	Number (%)
Age	
< 60	6 (42.9)
≥ 60	8 (57.1)
Sex	
Female	1 (7.1)
Male	13 (92.9)
Tumor stage	
III	6 (42.9)
IV	8 (57.1)
Tumor differentiation	
Well or moderate	6 (42.9)
Poor	8 (57.1)
Metastasis	
M1	9 (64.3)
M0	5 (35.7)
HER2_expression	
Negative	12 (85.7)
Positive	2 (14.3)
Lymph node involvement	
N3 stage	8 (57.1)
N0–N2 stage	6 (42.9)
HP-1 infection	
Negative	4 (28.6)
Positive	10 (71.4)
Drink	
Yes	3 (21.4)
No	11 (78.6)
Clinical prognosis	
PD	7 (50)
CR or SD	6 (42.8)
NE	1 (7.2)

### Cell-free DNA (cfDNA) extraction from plasma

Within two hours after collection of blood samples, the samples were centrifuged at 1600*g* for 10 min at 4 °C in tubes containing EDTA. After centrifuging the supernatants at 10,000*g* for 10 min at 4 °C, the plasma was collected and stored at 80 °C. Following the manufacturer's instructions, cfDNA was extracted from at least 3 mL plasma using the QIAamp Circulating Nucleic Acid kit (Qiagen). Qubit 2.0 Fluorometer and Qubit dsDNA HS kit (Thermo Fisher Scientific) were used to quantify cfDNA.

### Constructing the cfDNA library

To construct the cfDNA libraries, we used KAPA Library Preparation kit (Kapa Biosystems). Cleanup was performed with AMPure XP beads (Beckman Coulter), and DNA concentrations were quantified with the Qubit dsDNA HS Assay kit (Qubit 2.0 fluorometer). Target enrichment by hybridization was conducted using customized HapOncoCDx panel by HaploX (HaploX, Jiangxi, China) for cfDNA sequencing. Detailed instructions can be found in our previously published article (Wu et al. 2022). Sequencing of the libraries was carried out by HaploX (HaploX, Jiangxi, China) using an Illumina NovaSeq 6000 (Illumina).

### Helicobacter pylori antibody testing

Helicobacter pylori (HP) antibodies in serum were measured using Western blot analysis as described previously (Liu et al. 2020). All procedures were performed according to the manufacturer's instructions. The manufacturer's instructions were followed throughout. The presence of CagA and VacA zones at the same time or either of them appeared was considered to be HP-1 infection.

### PFS and OS analysis

Until cancer-induced death or dropout from the study, enrolled patients were followed up every 3–6 months after surgery. As of the end of the study, the time from the first day of patient treatment to disease progression or death was defined as PFS, whichever came first. From the day the patient first began treatment until the day of death or the last follow-up, OS was assessed. In terms of survival tests, we compared PFS and OS between ctDNA-positive and ctDNA-negative groups using Kaplan–Meier analysis. The definition of ctDNA-positive patients follows the following principles: during the postoperative follow-up period, we use the last blood specimen collected for sequencing analysis and define it as ctDNA positive if a ctDNA mutation is detected at that point; otherwise, it is defined as ctDNA negative.

### Data analysis

For the data analysis method generated by NGS-Panel, please refer to our previous article (Chen et al. 2019). We performed mutation calling using the Mutect2 tumor-only mode, with mutation filtering based on the criteria of DP < 5.0, QD < 2.0, or FS > 60.0. The SNV and indels were filtered as follows, (1) filter all variants with population frequency > 1% in ESP6500 and 1000 genomes database, (2) include somatic variants with variant allele frequency (VAF) ≥ 5%, sequence reads in support of the variant call ≥ 3, (3) filter mutations located in repetitive regions of the genome, SSRs, and (4) filter benign mutations. In addition, we have included a table (Supplementary Table 2) that provides detailed information on the variants detected for each sample, including the location, nucleotide change, VAF, positive/negative supporting read counts, and total read counts.

## Results

### Basic clinical characteristics of GC patients

The results of this study were based on 14 GC patients, and their clinical characteristics were shown in Table 1. Eight of them were over 60 years old and 13 were men. All pathotypes were adenocarcinomas, and nine patients were diagnosed with M1 metastasis. 12 and two patients were negative and positive in HER2 expression, respectively. 10 and four patients were positive and negative for HP-1 bacterial infection, respectively. The tumor grades of eight patients were poor differentiation. Half of the patients in the cohort progressed, and eight patients were in stage N3 according to lymph node staging.

### Somatic mutation spectrum and mutation signature of GC patients

**Fig. 1:**
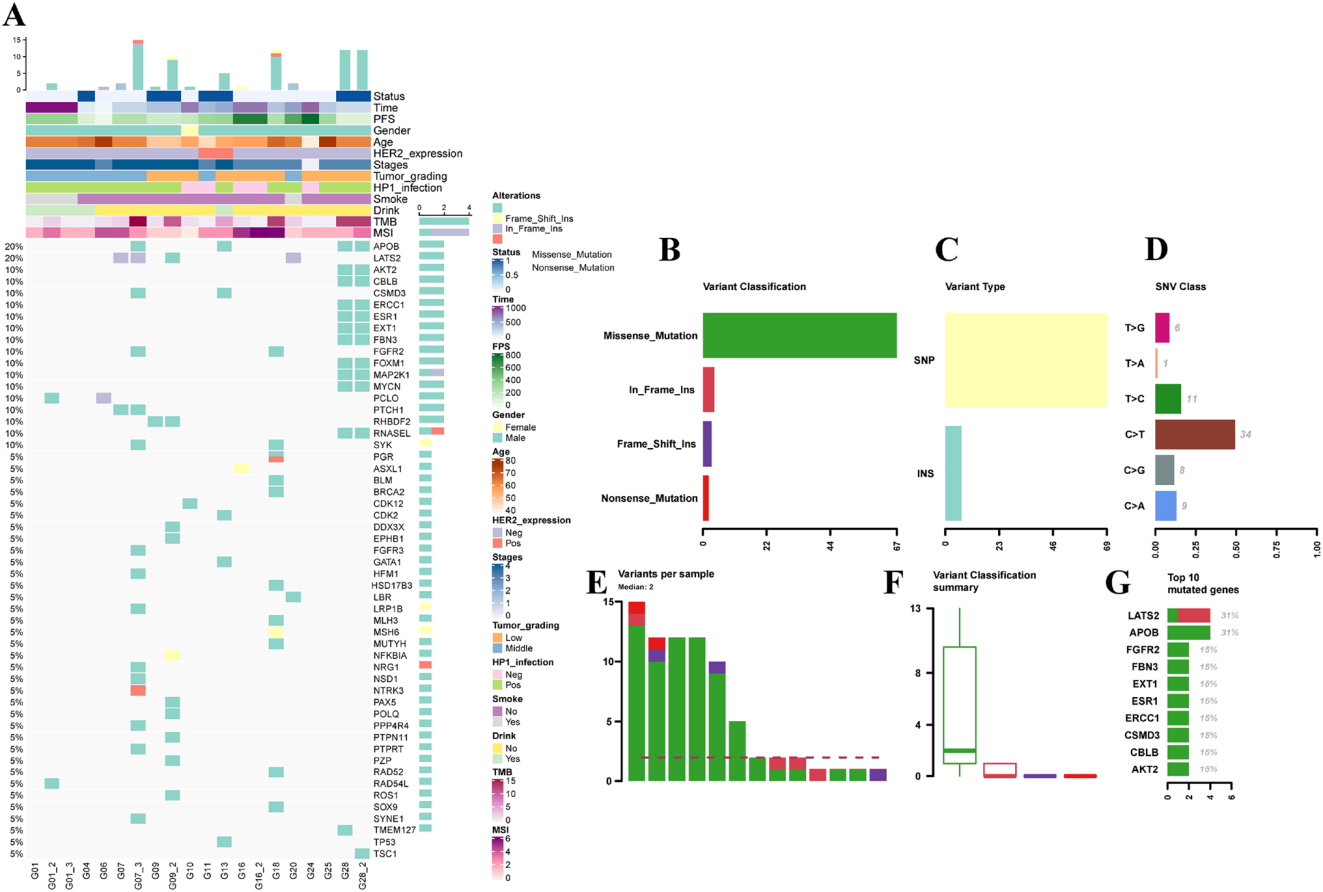
The somatic mutation landscape in GC patients. **A** An assessment of the genes and the frequency of ctDNA mutations in 20 plasma samples. **B**–**G** The landscape of GC patient mutations

Detection of ctDNA mutations and bioinformatics analyses were performed on 20 plasma samples obtained from 14 patients with GC. The landscape of their somatic mutations and signatures was plotted (Fig. 1). The cascading diagram showed the detected genetic mutations of ctDNA and clinical characteristics of GC patients (Fig. 1A). 53 genes with high-frequency mutations were identified in the 20 samples. The frequencies of mutation rates (VAF) range from 2 to 51%. After surgery, 8 patients had consistently detectable ctDNA mutations, and the remaining 6 patients had undetectable ctDNA mutations. Mutations of the missense type were most prevalent in GC patients (Fig. 1B). A single nucleotide variation (SNV) that was very common in GC was C>T (Fig. 1C, D). As seen in Fig. 1E, F, the mutation rates of each sample are shown. The top ten highly mutated genes were APOB (31%), LATS2 (31%), FGFR2 (15%), FBN3 (15%), EXT1 (15%), ESR1 (15%), ERCC1 (15%), CSMD3 (15%), CBLB (15%) and AKT (15%) (Fig. 1G).

### Correlation between ctDNA mutations and the clinicopathologic features of gastric cancer

**Fig. 2:**
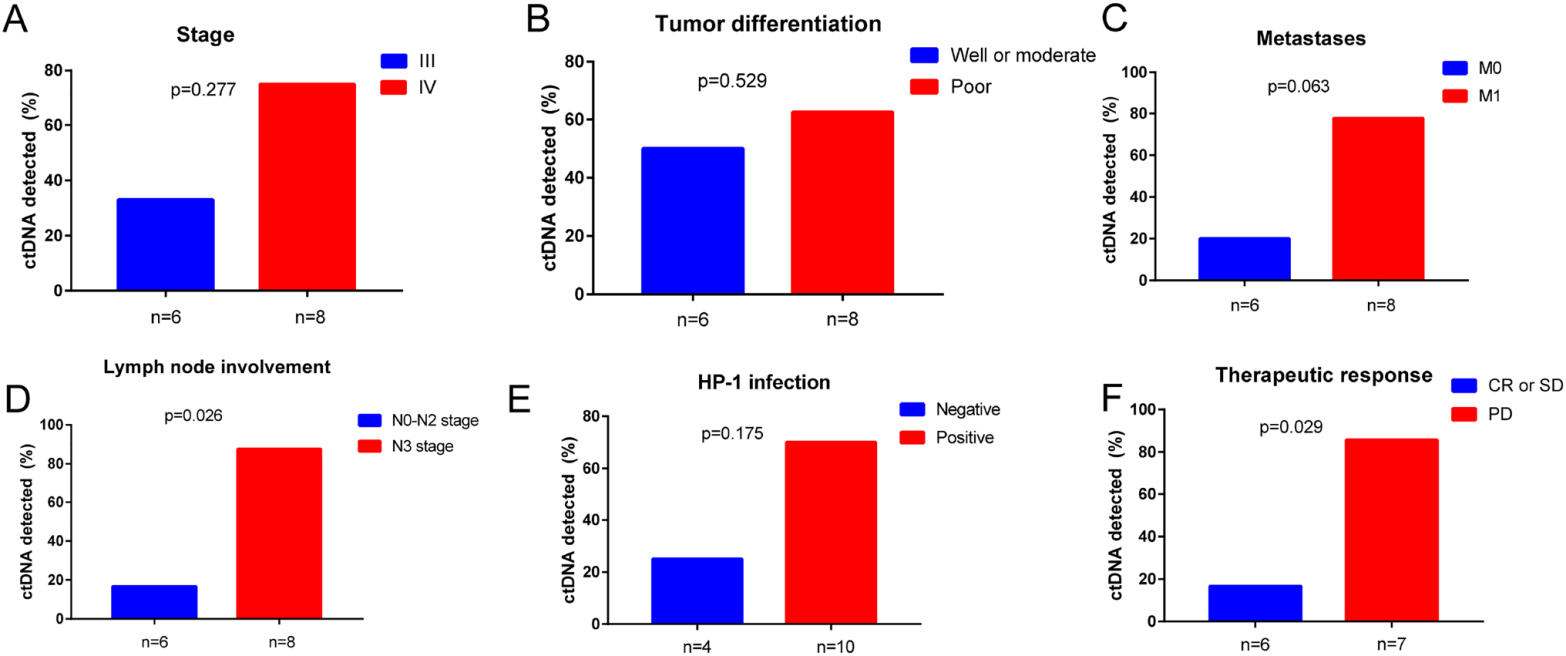
Correlation between ctDNA mutation detection and clinicopathological features of gastric cancer. **A** Stage (III vs IV). **B** Tumor differential (well or moderate vs poor). **C** Metastases (M0 vs M1). **D** Lymph node involvement (N0-N2 vs N3). **E** hp-1 infection (negative vs positive). **F** Therapeutic response (CR or SD vs PD)

Different clinicopathological characteristics were used to divide the patients into groups, and the correlation between the detection of ctDNA mutation and the clinicopathological features of gastric cancer was explored by Fisher's exact test. Like previous studies, ctDNA-positive patients in our cohort tended to have a poor prognosis. The detection rate of ctDNA in patients with stage IV cancer was 75% (6/8, Fig. 2A, P = 0.277), while in patients with stage III cancer, it was only 33%. Metastasis also seemed to be associated with ctDNA detection (6/8, Fig. 2C, P = 0.063), but in our cohort, tumor differentiation was not associated with ctDNA detection (Fig. 2B, P = 0.063). In addition, the detected rate of ctDNA mutation was significantly higher in patients with the N3 stage (Fig. 2D, P = 0.026) and patients with disease progression (Fig. 2F, P = 0.029) during treatment. It was also found that ctDNA detection tended to be positive in HP-1 infection patients (Fig. 2E, P = 0.175). In univariate analysis, tumor differentiation, staging (T staging, N staging, M staging), HP-1 infection, and treatment response were considered, which were consistent with Fisher's exact test results. Only T staging (Table 2, P = 0.02) and therapeutic response (Table 2, P = 0.027) were associated with ctDNA detection.

**Table 2:** Univariate analysis to determine the predictors of ctDNA detection in plasma sample

Clinicopathological variables	Univariable analysis	
	OR (95% CI)	P-value
Differentiation	0.511	0.641
Stage	1.791	0.132
T-stage	0.336	0.826
N-stage	3.555	0.020*
M-stage	2.639	0.055
Hp1-infection	1.946	0.148
Therapeutic	3.401	0.027*

### ctDNA mutations can predict the therapeutic response and be used for prognosis in gastric cancer

**Fig. 3:**
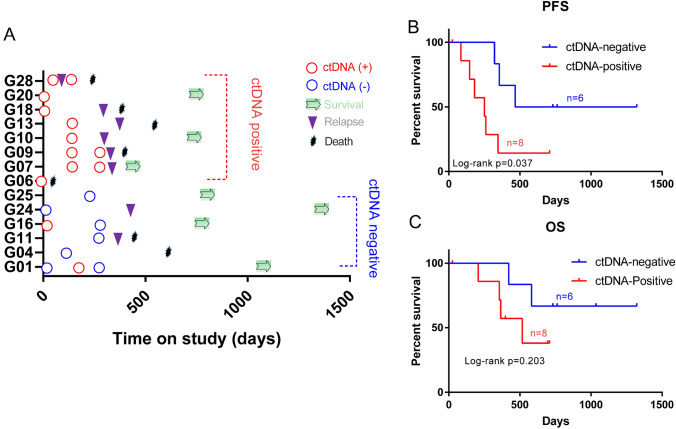
Detection of ctDNA mutation predicts patient survival. **A** The detailed survival data of the patients. ctDNA + : ctDNA-positive patients; ctDNA − : ctDNA-negative patients. Relapse means that the patient's condition is progressing. **B** Kaplan–Meier curves of PFS. **C** Kaplan–Meier curves of OS

Through long-term follow-up of patients, after a period of treatment, most ctDNA-negative patients survived (4/6), and only a few of them relapsed (2/6). In contrast, ctDNA-positive patients generally had disease progression (6/8), and more than half of them passed away due to disease progression (4/7, G06 died of myocardial infarction, Fig. 3A). The survival time of ctDNA-negative patients is almost always longer than that of ctDNA-positive patients. The PFS and OS of these two groups of patients were analyzed (Log-rank test). The results showed that the PFS time of ctDNA-negative patients was significantly increased (P = 0.037, HR = 3.578 95% CI 0.894–13.14, Fig. 3B) and the OS time was also longer (P = 0.203, HR = 2.931 95% CI 0.5567–14.34, Fig. 3C).

### Potential applications of ctDNA detection in monitoring gastric cancer

**Fig. 4:**
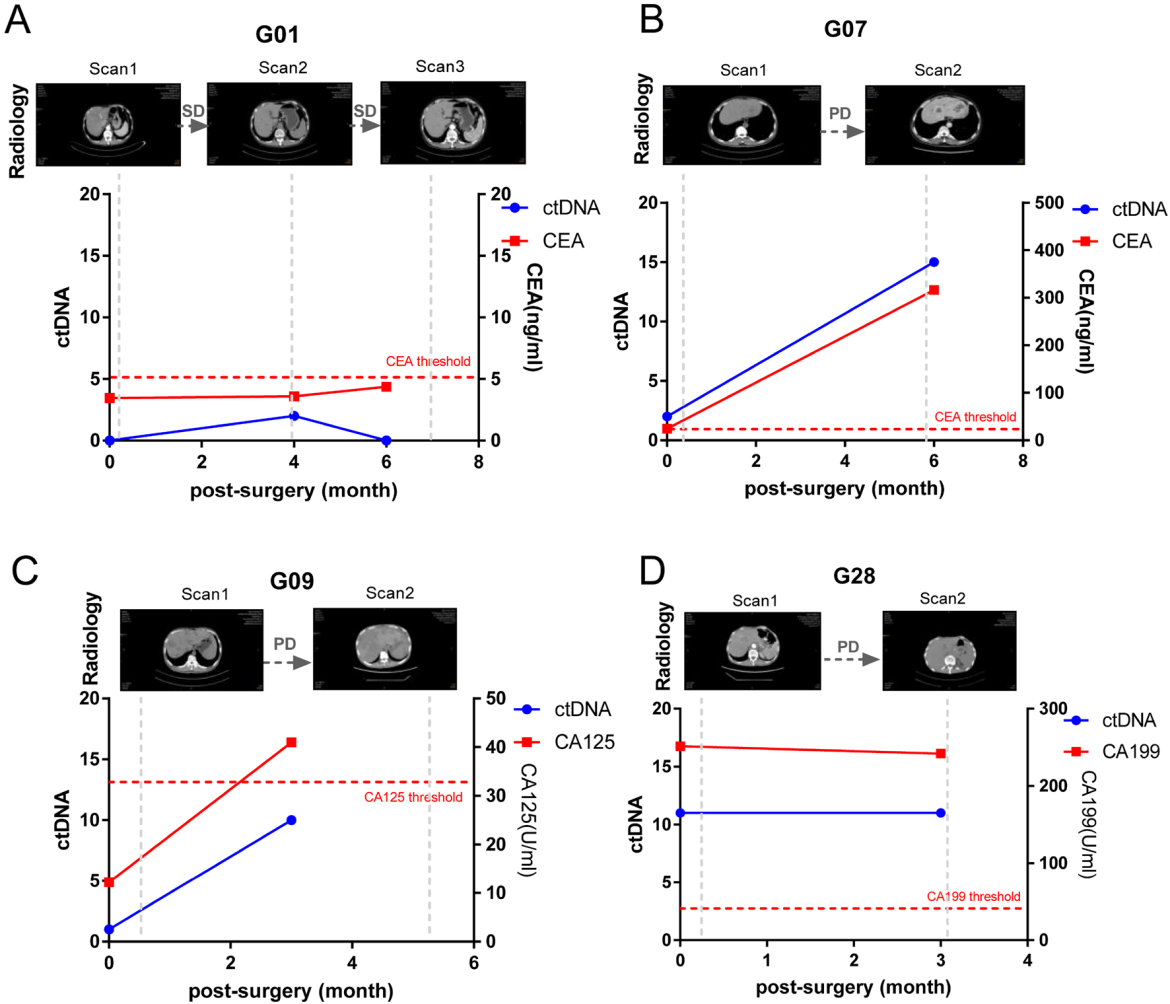
Application of ctDNA detection in prognosis monitoring of gastric cancer patients. Dynamic changes of ctDNA, radiology, and patient plasma tumor markers in the temporal dimension of four patients. *PD* progressive disease, *SD* stable disease. The red dotted line represents the threshold level of serum tumor markers in gastric cancer patients

In our patient cohort, a combined analysis of plasma samples and radiology collected at longitudinal postoperative time points was performed on four patients to assess the clinical utility of ctDNA as a biomarker for gastric cancer prognosis monitoring. From the radiology, patient G01 whose disease did not progress (Fig. 4A), had only a few genetic mutations in the second ctDNA detection, and ctDNA mutations were detected neither in the first nor the third-time point. Among the three patients with progressive disease, two patients, G07 and G09, had a rapid rise in the second time point with detected ctDNA mutations (Fig. 4B, C). The number of mutations in G28 patients was maintained at a high level (Fig. 4D). Plasma tumor markers CA199, CA125, and carcinoembryonic antigen (CEA), which are clinically used biomarkers for evaluating gastric cancer patients, were also used for combined analysis with ctDNA mutations (Fig. 4). In the G01 patient, his plasma CEA level remained below the normal threshold, whereas in two patients with increased ctDNA mutations (G07, G09), the plasma CEA levels in G07 and the plasma CA125 levels in G09 increased rapidly. In G28, it was reflected in the plasma CA199 which was far exceeding the normal level. These results validated that ctDNA detection can help clarify suspected radiological or plasma tumor markers and improve the accuracy of gastric cancer disease surveillance.

### CBLB mutation associated with prognosis of GC patients

**Fig. 5:**
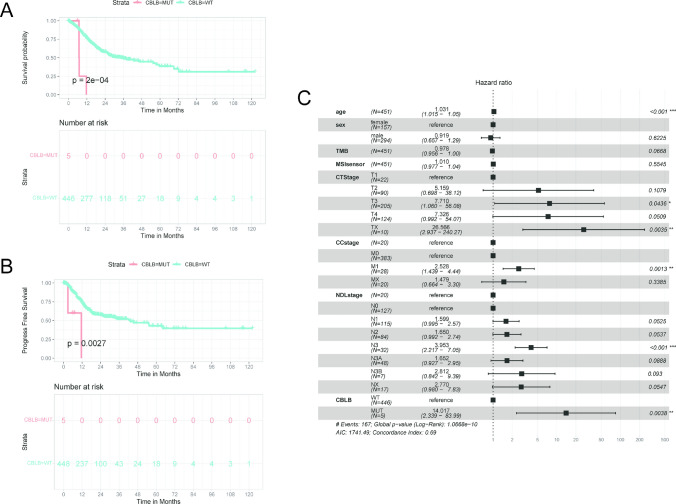
Relationship between CBLB mutation and prognosis of GC patients. **A** Survival analysis to explore the OS of GC patients between the wild type and CBLB mutation. **B** Survival analysis to explore the PFS of GC patients between the wild type and CBLB mutation. **C** CBLB mutations are associated with higher hazard ratio for gastric cancer patients

The top 10 genes with the highest mutation frequency were obtained. Mutation data from a large cohort of GC patients in the TCGA database were tested as the control to determine whether some mutations in the top 10 genes are potential prognosis biomarkers. A total of 451 gastric cancer patients were included in the TCGA database, out of which 14 patients were identified as carrying CBLB mutations. Among these patients, nine exhibited synonymous mutations, while the remaining five were detected with missense mutations (Supplementary Table 1). Our result showed that CBLB mutations were significantly different in overall OS, PFS analysis, and forest plot. The mutation frequency of the GC cohort in the TCGA database was very low (less than 2%) (Fig. 5C). However, the mutation frequency of CBLB in this study was high (15%) (Fig. 1G). Patients with CBLB mutations had significantly shorter OS and PFS than those with wild-type CBLB, too, according to a Kaplan–Meier analysis (P < 0.01) (Fig. 5A, B). Compared to the wild type, patients with CBLB mutations presented a significantly higher hazard ratio, which was 14 times higher than the wild type (P < 0.01) (Fig. 5C). And in this patient cohort, patients with CBLB mutations progressed more rapidly and most died within a year. After receiving 8 cycles of chemotherapy (oxaliplatin combined with 5-fluororacil and folinic acid), the prognosis was very poor, OS less than seven months, and PFS less than three months.

## Discussion

The global burden of GC remains considerable, as it is the third leading cause of cancer-related death. The prognosis for GC remains poor despite recent advances in systemic treatment (Ooki and Yamaguchi 2021). A subset of patients will eventually die from local recurrence, peritoneal recurrence, or distant metastasis (Songun et al. 2010), with a 5-year OS rate of 5–20% (Wagner et al. 2017). Current methods for assessing the risk of disease recurrence mostly rely on radiographic examinations (Becker et al. 2003), traditional blood biomarker screening, and imaging techniques to capture postoperative minimal residual disease (MRD) status (Smyth et al. 2016). However, these techniques all have different limitations, such as the inability to perform tumor regression grading and low sensitivity, therefore, limiting their use in clinical practice (Langer and Becker 2018; Leal et al. 2020). At present, with the continuous maturity of liquid biopsy technology, more evidence has shown that this tool has good clinical application potential (Alix-Panabières and Pantel 2021). For example, by detecting ctDNA, patients who may benefit from adjuvant chemotherapy can be identified, and MRD or preclinical metastasis can be detected earlier and more dynamically (Christensen et al. 2019; Qiu et al. 2021). Several studies have reported the clinical value of MRD detection and longitudinal disease surveillance in gastric cancer treatment. and for predicting the associated efficacy of neoadjuvant chemotherapy (Nakamura et al. 2021; Yang et al. 2020). However, there are few reports about the use of ctDNA to predict the prognosis of patients.

In this study, a total of 14 patients diagnosed with advanced gastric cancer (GC) were prospectively enrolled to assess the clinical utility of circulating tumor DNA (ctDNA) in predicting surgical prognosis. Due to certain constraints, only ctDNA samples were obtained and sequenced, while peripheral blood mononuclear cell (PBMC) samples were not sequenced. Consequently, concerns arise regarding the accurate identification of germline variants in the patients. To address this issue, population frequency analysis was employed as the primary filtering method during the second-generation sequencing analysis. This approach is widely recognized as the most crucial filtering technique due to the prevalence of population non-pathogenic polymorphisms in sequencing results, with more than 99% of such variants being directly eliminated using a high-quality reference population frequency database (Sudmant et al. 2015).

The selection of causal variants based on their frequency in unselected individuals is a critical step in both the data analysis process and the identification of potential causal variants. The efficacy of this screening process relies on the size and diversity of the reference population dataset employed (Lek et al. 2016). In our study, we utilized the ESP6500 and 1000 Genomes databases as references for filtering relevant variants (Foley et al. 2015; Zhang et al. 2015). By employing the filtering technique elucidated in the preceding data analysis, we endeavored to eliminate germline mutations to the greatest extent feasible. Our firm conviction lies in the efficacy of these analytical methodologies in ameliorating the prevalence of commonplace germline variants among the study cohort, consequently reinforcing the veracity of our findings.

We found that the positive ctDNA detection was significantly associated with worse clinical T stage and clinical therapeutic response in GC patients. This is similar to the conclusion of a previous breast cancer study (Zhou et al. 2019) that the positive ctDNA detection was significantly associated with increased metastasis and disease progression. Through long-term follow-up of patients, we found that ctDNA-positive patients were more likely to relapse, and also have poorer OS (P = 0.203) and PFS (P = 0.037). We speculate that the reason for the insignificant trend in OS could be that ctDNA-negative patients were mostly alive at the end of follow-up, resulting in more censored data in statistics. Conversely, in the PFS statistics, ctDNA-positive patients usually progressed.

Postoperative MRD and micrometastatic recurrence cannot be detected using routine clinical radiography and serum biomarkers. In GC patients, serum biomarkers such as carcinoembryonic antigen (CEA) and cancer antigen-199 (CA-199) can only detect about 40% of recurrences (Baiocchi et al. 2014; Căinap et al. 2015). Our study was conducted on four gastric cancer patients to evaluate the effectiveness of ctDNA as a biomarker for prognosis monitoring. One patient had few ctDNA mutations and no progression, while the other three patients showed a rapid rise in ctDNA mutations and disease progression. Plasma tumor markers CA199, CA125, and CEA were also used in combination with ctDNA mutations. In the patient with no disease progression, the plasma CEA level remained normal, while in the other three patients, plasma levels of CEA, CA125, and CA199 increased rapidly. Research has demonstrated that ctDNA analysis is a more sensitive technique for detecting tumor-specific genetic mutations than traditional biopsy methods or the assessment of typical cancer biomarkers used in clinical settings (Zhang et al. 2022). Notably, due to tumor heterogeneity, ctDNA analysis has a higher detection rate for FGFR2 amplification than tissue biopsy, which can enhance the effectiveness of treatment (Jogo et al. 2021). In lung cancer treatment, standard imaging methods used in routine clinical monitoring can only detect macroscopic disease recurrence, which can be confounded by inflammation or fibrosis, especially after radiation therapy (Ettinger et al. 2017; Huang et al. 2012). However, Chaudhuri et al. discovered that ctDNA testing shortly after treatment can identify patients with localized lung cancer who are at high risk of recurrence (Abbosh et al. 2018). Additionally, ctDNA testing after treatment may surpass standard imaging methods in monitoring patients for disease recurrence. In our study, the combined analysis of longitudinal time-point ctDNA, radiological, and serum biomarkers in four patients showed that ctDNA detection can be a good complement to radiological or plasma tumor markers, thereby improving the accuracy of gastric cancer prognosis monitoring.

In our analysis of the top 10 genes with the highest ctDNA mutation frequency in our cohort, we found that the CBLB gene, on the contrary, has very low mutation frequency in TCGA. In previously studies, mutations in the CBLB gene may lead to breast cancer (Cortes-Urrea et al. 2020) and associated with colorectal cancer metastasis (Ishaque et al. 2018). In the Kaplan–Meier survival curves, the OS and PFS for patients with CBLB mutations were significantly shorter (P < 0.01), and the hazard ratio was also much higher than that of the wild-type patients, suggesting that the CBLB mutations may be related to the prognosis of GC patients.

Nevertheless, the current study has certain limitations. For example, the role of ctDNA in predicting prognosis is prone to bias due to our small sample size. In the longitudinal study, samples were not collected from the same patient at longer intervals.

## Conclusion

In this study, we demonstrated that postoperative monitoring of ctDNA in GC patients can be used for patient prognosis. The positive detection of ctDNA in patients after surgery indicates that the disease is likely to progress and metastasize, and ctDNA-positive patients have worse PFS and OS. The combined detection of ctDNA, radiological, and serum biomarkers can effectively improve the accuracy of postoperative surveillance of gastric cancer. We also found that the mutations in CBLB gene may be a biomarker for the poor prognosis of gastric cancer patients. This small cohort study demonstrated the utility and feasibility of ctDNA in gastric cancer prognosis monitoring.

## Supplementary Information

Below is the link to the electronic supplementary material.

Supplementary file1 (XLSX 10 kb)

Supplementary file2 (XLSX 17 kb)

## Data Availability

The data that support the findings of this study are available on request from the corresponding author. The data are not publicly available due to privacy or ethical restrictions.
